# A Review of Research Progress on Machining Carbon Fiber-Reinforced Composites with Lasers

**DOI:** 10.3390/mi14010024

**Published:** 2022-12-22

**Authors:** Junke Jiao, Xiangyu Cheng, Jiale Wang, Liyuan Sheng, Yuanming Zhang, Jihao Xu, Chenghu Jing, Shengyuan Sun, Hongbo Xia, Haolei Ru

**Affiliations:** 1 School of Mechanical Engineering, Yangzhou University, Yangzhou 225009, China; 2 PKU-HKUST ShenZhen-HongKong Institution, Shenzhen 518057, China; 3 School of Mechanical and Vehicle Engineering, Linyi University, Linyi 276005, China; 4 Ningbo Institute of Materials Technology and Engineering, Chinese Academy of Sciences, Ningbo 315201, China

**Keywords:** laser machining, carbon fiber-reinforced composites, process optimization, heat affected zone, numerical simulation

## Abstract

Carbon fiber-reinforced composites are widely used in automobile, aerospace and military lightweight manufacturing due to their excellent mechanical properties such as light weight, excellent fracture resistance, corrosion resistance and wear resistance, etc. However, because of their high hardness, anisotropy and low interlayer strength characteristics, there are many problems with machine carbon fiber-reinforced composites with traditional methods. As a non-contact processing technology, laser machining technology has lots of advantages in carbon fiber-reinforced composites processing. However, there are also some defects produced in laser machining process such the heat affected zone, delamination and fiber extraction due to the great difference of physical properties between the carbon fibers and the resin matrix. To improve the quality of carbon fiber-reinforced composites laser machining, lots of works have been carried out. In this paper, the research progress of carbon fiber-reinforced composites laser machining parameters optimization and numerical simulation was summarized, the characteristics of laser cutting carbon fiber-reinforced composites and cutting quality influence factors were discussed, and the developing trend of the carbon fiber-reinforced composites laser cutting was prospected.

## 1. Introduction

**Figure 1:**
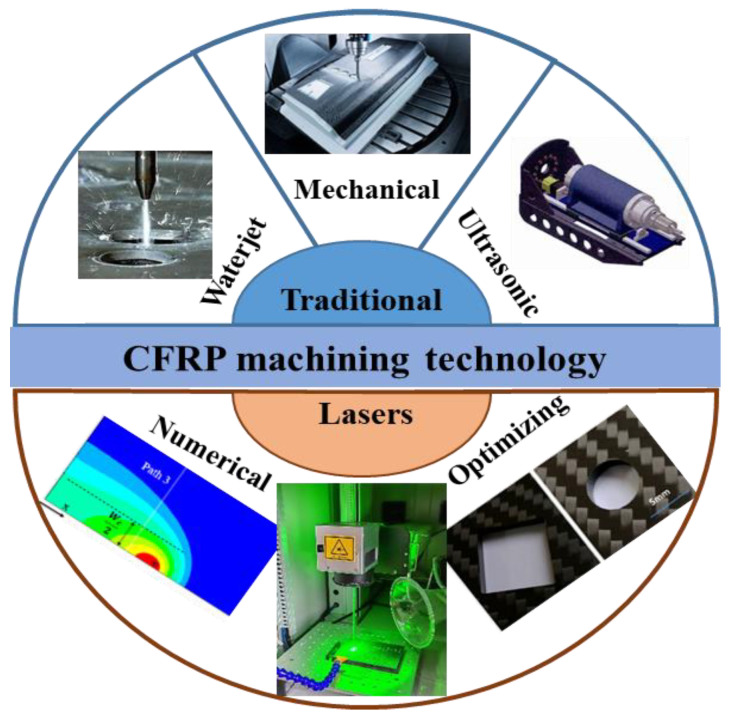
CFRP machining technology.

The carbon fiber-reinforced composites (CFRP) have the advantages of high specific strength, low density, light weight and have become a key material for the aerospace and new energy vehicle light-weight manufacturing [1,2]. CFRP machining is an important process in its application. The machining methods include mechanical and special processing technique as shown in Figure 1 [3,4,5,6].

**Figure 2:**
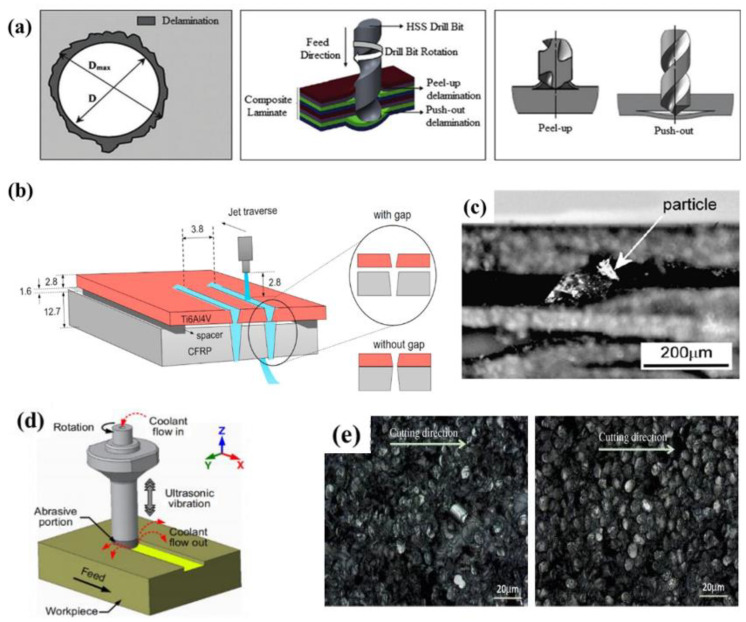
(**a**) Illustration of the delamination failure emerging as a result of drilling Reprinted/adapted with permission from Ref. [16]; Copyright 2018, Elsevier. (**b**) Experimental set up depicting AWJ machining of Ti6Al4V/CFRP stack Reprinted/adapted with permission from Ref. [21]; Copyright 2019, Elsevier. (**c**) Delamination produced in CFRP waterjet cutting process Reprinted/adapted with permission from Ref. [28]; Copyright 2008, Elsevier. (**d**) Illustration of rotary ultrasonic machining (RUM) surface grinding process Reprinted/adapted with permission from Ref. [31]; Copyright 2017, Springer Nature. (**e**) Uneven fiber fractures and interlayer fragmentation in CFRP ultrasonic machining Reprinted/adapted with permission from Ref. [33] Copyright 2014, Elsevier.

The mechanical machining includes cutting, drilling and milling, and the carbide tools are used in the machining process [7,8]. Koklu et al. [9] applied a new low-temperature processing method to the drilling of CFRP, immersed CFRP in liquid nitrogen, and directly processed it in low-temperature coolant. The machining characteristics such as thrust, delamination, tool wear, surface roughness and topography were compared with those under dry conditions. The experimental results showed that the low temperature processing method can improve the process-ability of CFRP. Morkavuk et al. [10] studied the milling performance of CFRP in low-temperature medium. The results showed that the low temperature cooling makes the structure of the workpiece brittle, prevents the thermal damage of the machined surface, improves the chip’s crack resistance, and can obtain a smoother surface. Wang et al. [11] studied the influence of drilling area temperature on the material properties and quality of CFRP. The results showed that the temperature range of the optimum drilling area is lower than the glass transition zone temperature of CFRP composites, and higher than the brittle deformation of resins. Ali et al. [12] studied the cutting performance of tungsten carbide YG6X (WC-6 wt% Co) conventional double groove twist drill on multidirectional T700 CFRP plate and analyzed the influence of different cutting speeds and tool wear modes on drilling performance. Finally, a set of suitable drilling parameters is proposed. Under the conditions of cutting speed of 9000 rpm and feed rate of 400 mm/min, the best quality hole can be produced. The mechanical process is the common method in CFRP machining. However, the cutting force of such tools in the machining process is too large to produce defects such as burrs, delaminated tears and fractures [13,14,15] (Figure 2a), which influence the mechanical resistance of the CFRP components.

Besides the mechanical machining method, the special processing technology is also important method to machining CFRP, which mainly includes waterjet cutting, ultrasonic vibration auxiliary processing, laser processing, etc [16,17,18]. The waterjet cutting uses a high-pressure abrasive water jet for processing [19,20]. Compared with mechanical machining, the high-pressure water flow cutting technique is environmentally friendly. Pahuja et al. [21] evaluated the machinability of stacked titanium (Ti6Al4V) and CFRP by using the abrasive water jet (AWJ) processing technology. The results showed that the surface roughness and corner width change greatly under the condition of low jet power (Figure 2b). Kumar et al. [22] studied AWJ sections machined under two different process parameters. The results showed that fiber pullout and interlaminar tear can be observed in AWJ cutting under higher jet pressure. Alberdi et al. [23,24] studied the feasibility of machining CFRP/Ti6Al4V stack with AWJ under different process parameters. The final results showed that under almost all cutting conditions, Ti6Al4V has a positive cone angle. As mentioned above, waterjet cutting technology has a low processing cost and there is no tool wear and excess heat during process [25]. However, the high-pressure water jet will make the soaked polymer softer during processing, exacerbating the adhesion of the carbon fiber matrix [26,27] and the ability of the cutting surface to absorb moisture, resulting in defects in mechanical properties. Moreover, the impact of water cutting process on CFRP is obvious, making it susceptible to defects such as delamination, burrs, and tapers [28,29] (Figure 2c).

Ultrasonic vibration machining is another important CFRP machining method, it can reduce cutting force, reduce tool wear and improve machining quality [30]. Ning et al. [31] studied the surface grinding of rotary ultrasonic machining to process CFRP. It was found that the theoretical prediction trend is in good agreement with the experimental results on the relationship between the forward cutting force and the input variables (Figure 2d). Cong et al. [32] compared rotary ultrasonic machining with carbon fiber-reinforced plastic twist drill in terms of cutting force, torque, surface roughness, delamination, tool life, material removal rate, etc. The experimental results showed that rotary ultrasonic machining has advantages in almost all these aspects. At present, rotary ultrasonic machining is better used in milling and drilling, but when it is used for composite cutting, problems such as low efficiency, wide kerf, uneven fiber fracture and interlaminar fragmentation will occur [33], which will affect the performance and life of CFRP parts (Figure 2e).

**Figure 3:**
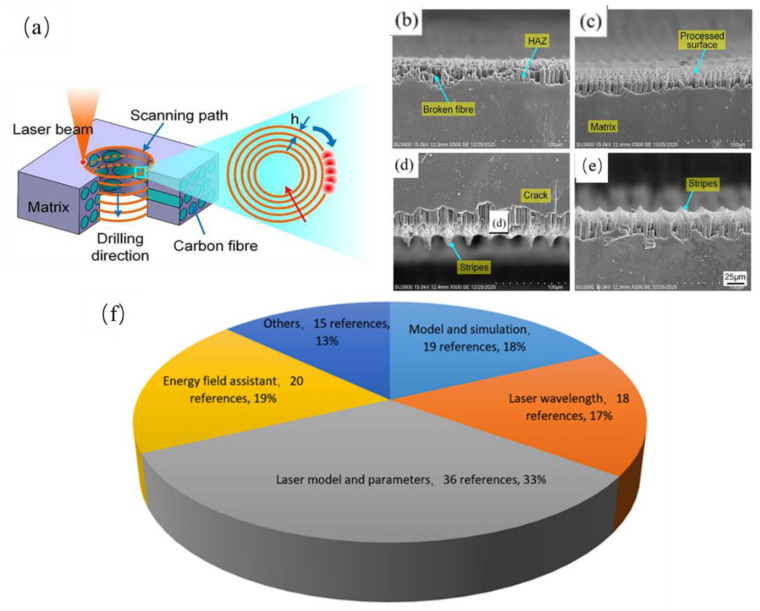
(**a**) Cutting hole in CFRP plate; (**b**–**e**) HAZ, crack, broken fibre produced in laser cutting process; Reprinted/adapted with permission from Ref. [40]; (**f**) Different aspects of CFRP laser machining. Copyright 2021, Elsevier.

As a non-contact processing technology, laser machining has lots of advantages in composites processing [34,35]. Compared with mechanical methods, there is no tool wear and cutting force in the machining process (Figure 3a). Laser cutting has good quality, high precision, narrow incision width and good surface roughness. Due to the anisotropy and non-uniform structure of carbon fiber composites, non-traditional processing technology will lead to material failure, such as fiber pullout, matrix cracking, delamination and expansion. During laser cutting, there is no contact with CFRP composite materials, no tool wear, and no need to replace the tool during processing. As long as the output parameters of the laser are changed, the noise is low, the vibration is small and there is no pollution during processing. Compared with ultrasonic-assisted processing and waterjet processing, laser processing will not produce impact damage or vibration damage. The efficiency can be improved by 20% and can machine CFRP components with various complex curves and shapes [36]. However, due to the large difference in the physical properties between the carbon fiber and the resin matrix, the resin matrix material produces ablation during the laser machining process, and the heat-affected zone (HAZ) [37], crack and broken fibre was produced, which seriously affects the processing quality of CFRP [38,39] (Figure 3b–e). In order to improve the quality of CFRP laser machining, lots of works about machining process parameters optimization and theoretical model analysis have been carried out. Laser scanning times, scanning speed and average power will affect the quality of laser processed parts. The number of scans is the number of cycles of part pattern processing, which determines the total number of pulses received and the heat absorbed by the CFRP composite surface processing area. More scanning times can play a more sufficient role in ablation and material removal of the processing area of CFRP composite and improve the processing accuracy. Scanning speed refers to the movement speed of the laser spot on the surface of CFRP composite materials during processing. It controls the number of pulse overlaps and the processing path and will affect the energy absorbed in the unit area of the sample surface processing area. The faster the scanning speed is, the fewer pulses are received in the unit area of the processing area, and the smaller the absorbed energy is. The average power of laser is also an important parameter that affects the processing quality of parts. Too large or too small average power will reduce the processing quality of parts. It should be adjusted reasonably according to the actual production and processing.

By reviewing papers on laser machining of CFRP, it was found that most of the investigations focused on machining parameters optimization and numerical simulation (Figure 3f). The laser machining parameters mainly include the laser wavelength, the laser mode, the laser power and the machining velocity, ect. The numerical simulation includes mathematical model established and numerical calculation of laser machining CFRP. In this work, the research progress of machining parameters optimization and numerical simulation was discussed, the characteristics of laser machining CFRP and machining quality influence factors were summarized, and the developing trend of the technology of CFRP laser machining was prospected.

## 2. CFRP Laser Processing Optimization

During CFRP laser machining, the energy to evaporate or sublimate carbon fibers is higher than that of the resin matrix [40,41]. Therefore, in the beam-material interaction process, the time vaporizing the carbon fibers is longer than that vaporizing the resin matrix. Before the carbon fibers are vaporized, a large amount of heat conducts through the carbon fibers and overheats the resin matrix. This heat makes the resin matrix experience thermal degradation and causes the fibers to peel from the substrate, which will lead to delamination [42,43]. Previous investigations show that these thermal defects can be minimized by optimizing the machining parameters such as the mode, the scanning speed, the laser power, the pulse characteristics and the wavelength of the laser [44].

### 2.1. Effect of Laser Wavelength on CFRP Machining Quality

**Figure 4:**
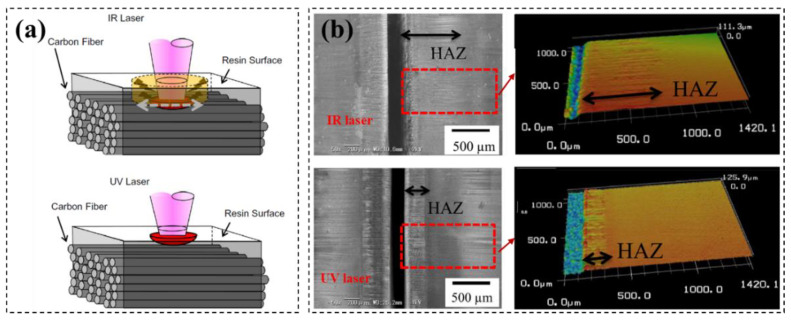
(**a**) Material removed mechanism for UV and IR lasers. (**b**) HAZ for CFRP cutting with IR and UV lasers Reprinted/adapted with permission from Ref. [46]. Copyright 2016, Elsevier.

CFRP has different absorption coefficients for lasers with different wavelength. To this end, the CFRP machining quality is different for different wavelength lasers [45]. Takahashi compared the effects of infrared (IR) and ultraviolet (UV) lasers on CFRP cutting quality experimentally [46]. For the IR laser, the polymer matrix absorption rate of laser is less than 15%, and 85% of the energy passes through the polymer matrix to heat the carbon fibers directly. On the other hand, the UV laser energy is absorbed by the resin matrix almost completely (Figure 4a). To this end, the UV laser has a better cutting edge quality compared to IR laser in CFRP cutting, and the HAZ is larger for IR laser than that for UV laser (Figure 4b). According to Xu’s research [47], the absorption rate of infrared light by carbon fibers is close to 80%, and the laser energy is mainly absorbed by carbon fibers, while the matrix is indirectly heated by hot carbon fibers, rather than the laser beam itself. On the other hand, in the ultraviolet wavelength range, the polymer matrix and fibers can absorb the laser well, infrared light can pass through the CFRP epoxy resin. Wolynski cut CFRP with IR and UV lasers and concluded that the cutting quality of ultraviolet light is better than that of IR laser [48]. Qi cut CFRP with 266 nm wavelength lasers and performed a multivariate linear regression analysis on the data, and the empirical formula of CFRP incision width and heat affected zone width was obtained, which provided a reference for laser parameter selection for CFRP laser cutting [49].

**Figure 5:**
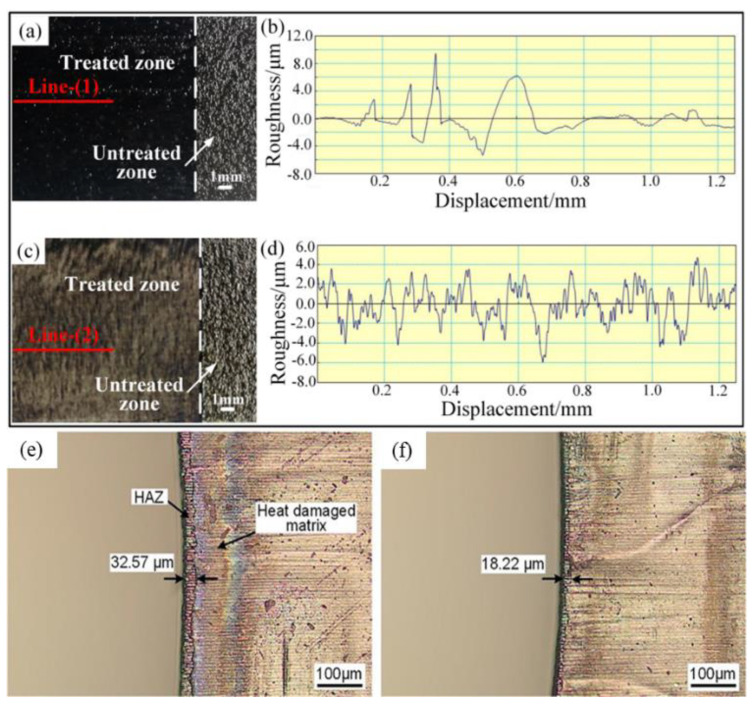
(**a**) macromorphology of IR laser cleaning; (**b**) surface roughness test result detected at marked line-(1); (**c**) macromorphology of UV laser cleaning; (**d**) surface roughness test result detected at marked line; Reprinted/adapted with permission from Ref. [50] Copyright 2019, Springer Nature. (**e**) HAZ with fill scan from inside out method with SSM; (**f**) HAZ with fill scan from inside out method with ISM (m ¼ 3) Reprinted/adapted with permission from Ref. [52]. Copyright 2021, Elsevier.

Yun et al. [50] studied IR laser surface treatment and UV laser surface treatment of CFRP laminates under different laser treatment parameters and analyzed their microstructure characteristics and the results of energy dispersive spectroscopy (EDS). The results showed that the thermal effect of IR laser leads to the combustion of carbon fibers, and the UV laser surface treatment without thermal effect ensures the integrity of carbon fibers (Figure 5a–d). Hong et al. [51] studied the IR laser surface treatment and peeling layer treatment of various laser processing parameters. The results showed that the average shear strength of the adhesive joint obtained by IR laser treatment is 20.158 MPa, while the average shear strength of the joint obtained by peeling layer treatment is 32.574 MPa. Yuan et al. [52] proposed a staggered scanning mode (ISM) based on the top-down default sequential scanning mode (SSM) of the multi-layer concentric circle filling scanning process, which improves the surface quality of CFRP plates through nanosecond ultraviolet laser drilling. Compared with SSM, the overall average HAZ width of ISMs is reduced by 25.85% by reducing the heat accumulation effect of adjacent tracks (Figure 5e–f). Yu et al. [53] controlled the scanning speed of UV laser, and the surface of CFRP was completely cleaned and partially cleaned. The results show that the main damage form of CFRP bonded joints obtained by complete and partial UV laser cleaning is mixed damage, in which interface damage and cohesive damage play an important role in the tensile properties of the two types of joints respectively.

### 2.2. Effect of Laser Mode and Parameters on CFRP Machining Quality

Both continuous wavelength (CW) laser and pulsed lasers have been applied to machine CFRP [54,55,56]. The CW laser has a high power and can remove material quickly, so the efficiency can be improved about 25%. However, the heat input into the material is large for the CW laser, which is easy to cause thermal damage. In order to reduce the thermal damage in machining CFRP with CW lasers, investigations have been carried out to optimize the machining process. Goeke [57] used CW fiber lasers to cut CFRP and found that both HAZ and seam width decreased significantly with increasing of the laser scanning speed. Klotzbach [58] also confirmed that the HAZ can be reduced greatly by increasing the laser scanning speed. Bluemel [59] studied the effect of machining speed on HAZ width with 6 KW of CW lasers and found that HAZ decreases with increasing of the cutting speed under the given laser power. Rao [60] used a 400 W CW laser to cut CFRP, and investigated the effect of laser power, beam scanning speed and auxiliary gas flow on the cutting quality by using the response surface method.

**Figure 6:**
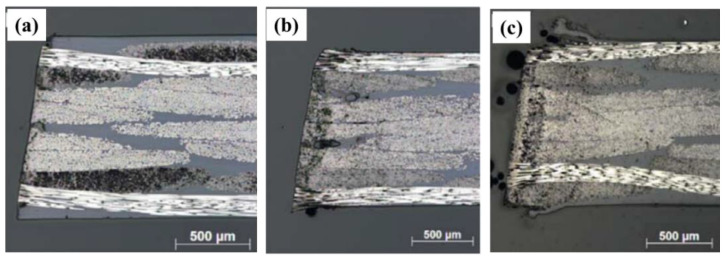
(**a**) Cutting CFRP with picosecond laser (laser power 50 W, Pulse duration 6 ps, cutting speed 15 m/min); (**b**) nanosecond laser (laser power 750 W, Pulse duration 30 ns, cutting speed 15 m/min); (**c**) CW lasers (laser power 4000 W, cutting speed 9.1 m/min). Reprinted/adapted with permission from Ref. [61]. Copyright 2014, Elsevier.

Bluemel [61] applied CW lasers and the pulsed lasers to cut CFRP, and found that the efficiency is higher than the pulsed laser about 25%. However, the short-pulsed laser has the smallest HAZ (Figure 6) and maximum tensile strength is larger than the CW lasers. Riviiro [62,63] used CO_2_ lasers with CW and pulsed mode to cut CFRP and studied the effect of processing parameters on incision width, HAZ and cross-sectional quality. Li [64] studied the cutting quality and cutting surface topography of CFRP laminates cutting by CW fiber lasers with single-channel and multi-channel processing paths.

**Figure 7:**
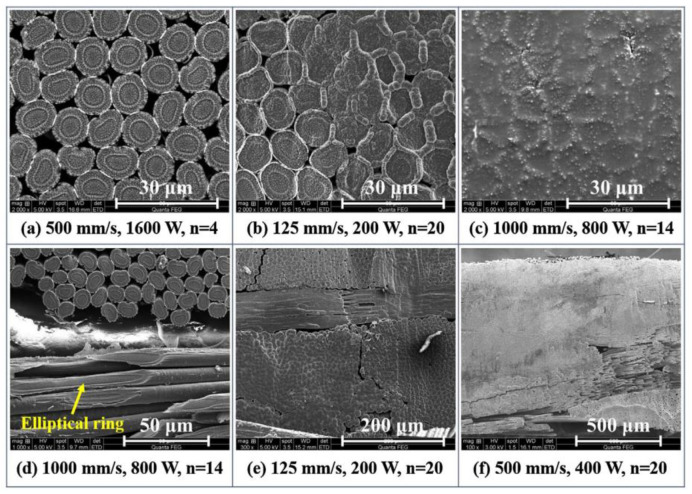
**(a**–**f)** SEM images of the cut surfaces, showing different degrees of fiber fusing Reprinted/adapted with permission from Ref. [65]. Copyright 2019, Elsevier.

Sehyeok et al. [65] studied cutting CFRP sheets using a 2 KW multimode fiber laser (Figure 7). The research results showed that with the increase in laser passing times, the corner width, the matrix evaporation width and the matrix recession width will also increase until cutting through, but once through cutting occurs, they will not change too much. In the process of laser irradiation, the pressure inside the corner edge increases significantly, and the carbon fibers at the corner edge can change into liquid, thus forming a molten section. Wei et al. [66] irradiated CFRP with a high intensity CW laser and proposed a tensile strength prediction model of orthogonal CFRP under CW laser irradiation. It was found that the prediction model can reflect the decreasing process of tensile strength under laser irradiation, especially in the case of high intensity laser irradiation and high tensile load.

**Figure 8:**
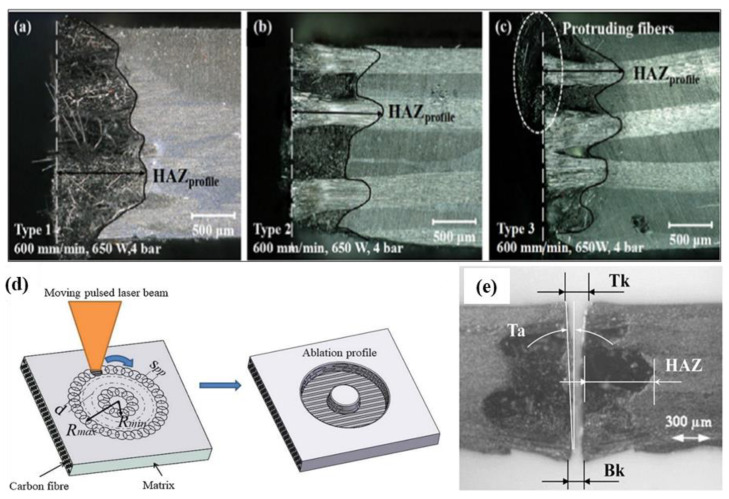
Optical image of cross section to the cut edge of samples with fiber orientation of (**a**) +45°/−45°, (**b**) 0°/90°, and (**c**) woven Reprinted/adapted with permission from Ref. [67]; Copyright 2018, Springer Nature. (**d**) Schematic description of pulsed laser coaxial-trepan drilling process by a single-beam Reprinted/adapted with permission from Ref. [68]; Copyright 2020, Elsevier. (**e**) Examples of kerf geometry Reprinted/adapted with permission from Ref. [69]. Copyright 2018, Elsevier.

Jun [67] cut a CFRP-laminated plate overlay structure with fiber lasers and adjusted the processing parameters to study the change of the surface quality of the laminated plate. The results showed that the HAZ level of laser cutting CFRP laminates is highly related to the fiber orientation, laser power and cutting speed (Figure 8a–c). Tao [68] studied and proposed a new double beam dislocation (DBOD) laser drilling technology (Figure 8d). The thickness of CFRP specimen reaches 10 mm by using DBOD process, which is significantly improved compared with other studies. Leone et al. [69] used 150W Nd: YAG pulse laser to cut 1 mm thick CFRP plate. It is found that the laser used can cut CFRP plates with a maximum speed of 12 mm/s (Figure 8e).

**Figure 9:**
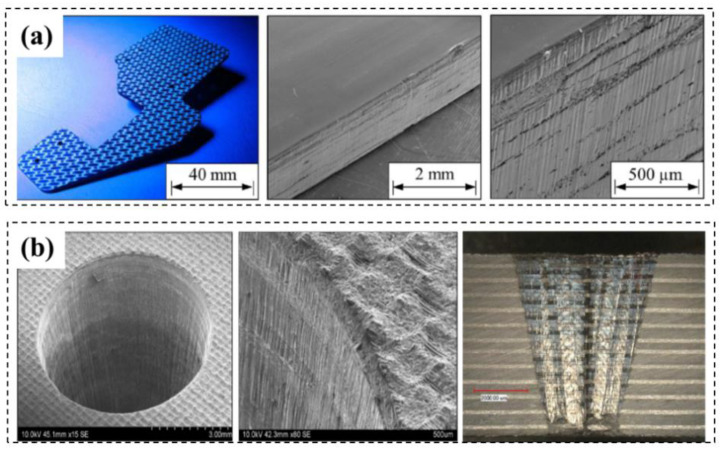
(**a**) Cutting CFRP with nanosecond laser (max.7mJ@10 kHz, 30 ns) Reprinted/adapted with permission from Ref. [76]; Copyright 2022, Elsevier. (**b**) drilling 6 mm diameter hole on a 6 mm thickness CFRP plate with picosecond laser (21 W, 0.5 MHz, 10 Ps) Reprinted/adapted with permission from Ref. [77]. Copyright 2016, Springer Nature.

The irradiation time of each pulse is very short for the ultrashort pulse laser relative to the CW laser and the long-pulsed laser. So, there is a sufficient cooling time between two pulses and cutting profile with less thermal damage can be obtained [70,71,72,73]. In CFRP cutting with pulsed lasers, the process parameters such as the pulse width, the frequency, and the processing path are the key parameters affecting the cutting quality. Freitag [74] optimized the pulse energy and the repetition rate of the pulsed laser to reduce the width of the HAZ and found that a high cutting quality can be obtained with a high pulse energy and high repetition rate at the same average laser power. Leone [75] cut CFRP with a Yb:YAG pulsed laser, and it was found that a better cutting quality was obtained with multi-channel technology at a higher scanning speed, and the width of HAZ can be reduced significantly. He also [69] used the Nd:YAG laser to cut CFRP and investigated the influence of process parameters on the geometry of the cutting slit and the HAZ at a speed of 12 mm/s. Yang [76] monitored the quality of nanosecond UV laser processing using acoustic emission technology and found that the CFRP laser cutting acoustic emission signal is consistent with the kerf width in both time and frequency domains (Figure 9a). Salama [77] drilled CFRP composites using a 400 W picosecond laser, and studied the influence of laser power, repetition rate, and scanning line distance on material removal efficiency and HAZ. A through-hole with diameter of 6 mm was drilled on a 6 mm thickness CFRP plates using scanning galvanometer ring cutting technology, and it was found that the laser inlet had a small HAZ (<25 μm), but the hole taper angle is larger, which is about 15°, and the processing time is about 3 min (Figure 9b).

**Figure 10:**
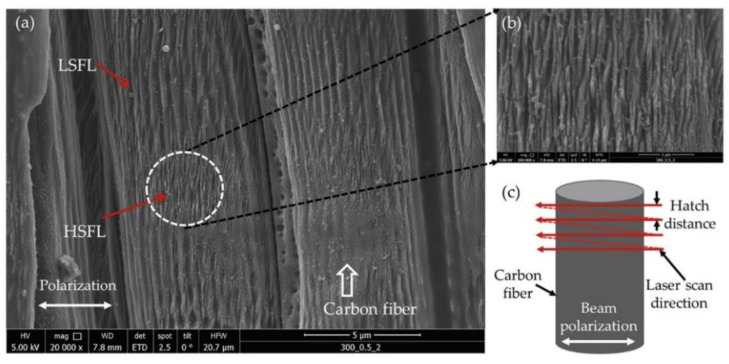
(**a**) Formation of laser induced periodic surface structure on carbon fiber surface (**b**) high spatial frequency laser induced periodic surface structure (HSFL)micrograph, and (**c**) schematic of scanning and beam polarization direction Reprinted/adapted with permission from Ref. [80]. Copyright 2020, Elsevier.

Li et al. [78] used a 532 nm nanosecond fiber laser with adjustable pulse duration to drill holes in the CFRP plate. It was found that under the condition of short pulse duration, the HAZ width was narrow, the minimum HAZ width at the edge of the hole inlet was 18.74 µm, and the matrix material near the HAZ was less damaged. Ma et al. [79] used 1064 nanosecond pulse laser to cut CFRP and GFRP materials and compared the propagation speed and breakdown time of the plasma plume. Kumar et al. [80] use a femtosecond laser system to generate the wettability transformation of the surface of CFRP with micro/nano structures. It was found that laser induced periodic surface structure was realized on carbon fiber surface at different defocusing distances and pulse energy (Figure 10).

**Figure 11:**
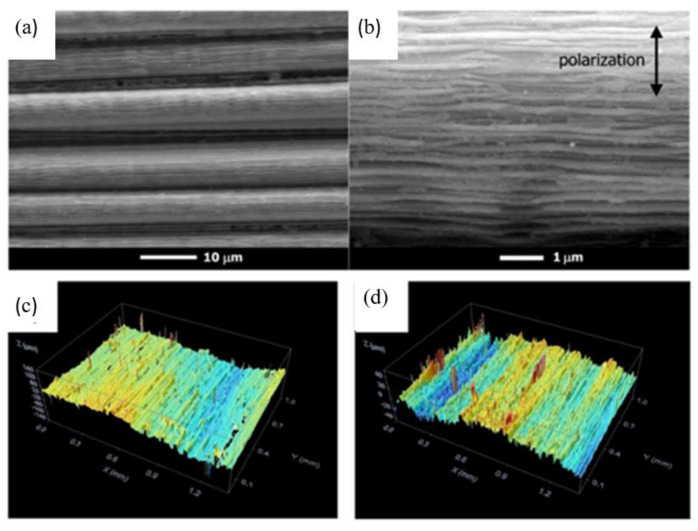
(**a**,**b**) SEM images of the central region of laser tracks produced with 0.35 mJ and 5 mm/s Reprinted/adapted with permission from Ref. [83]; Copyright 2017, Elsevier. (**c**,**d**) Pictures showing surface topography of GFRP Reprinted/adapted with permission from Ref. [84]. Copyright 2013, Elsevier.

Freitag et al. [81] proposed an approximate analytical expression for the minimum feed rate required to avoid evaporation of the matrix material during the ultra-short pulse processing of CFRP. It was found that the residual heat is related to the pulse energy and pulse repetition rate. Hu et al. [82] used a 355 nm emitting Nd: YVO4 picosecond pulsed laser system to study the effects of milling parameters on surface quality and material removal rate. The results showed that the laser grinding process of CFRP is complex, and the optimized laser power, grinding speed and hatch distance are 11.76 w, 2200 mm/s and 0.015 mm, respectively. Oliveira et al. [83] used a femtosecond laser with a wavelength of 1024 nm and a duration of 550 fs to treat unidirectional carbon fiber-reinforced epoxy matrix composites. The results showed that a selective removal of epoxy resin and exposure of carbon fibers can be achieved by using appropriate processing parameters (Figure 11a,b). Dittmar et al. [84,85] studied the effects of pulse overlap, focus diameter and the resulting flux on process quality and processing time. The results showed that the nanosecond pulsed UV laser can process two kinds of fiber-reinforced composites and achieve good surface quality without burn marks or other thermal damage areas (Figure 11c,d).

**Figure 12:**
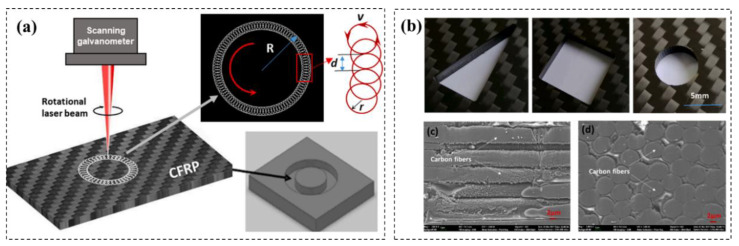
(**a**) CFRP high-speed laser rotary cutting technology with picosecond laser; (**b**) cutting CFRP for different shapes;(**c**,**d**) Micro schematic diagram after cutting. Reprinted/adapted with permission from Ref. [90]. Copyright 2021, Elsevier.

Jiang [86] conducted CFRP cutting experiments using picosecond lasers and studied the influence of laser parameters on cutting quality. The results showed that, when the cutting direction was parallel with the upper carbon fibers, the HAZ is minimal, and when the cutting direction is perpendicular to the upper carbon fiber arrangement, the HAZ is maximum. The HAZ increases with the increasing of the repetition rate, and the cutting efficiency increases 15% firstly and then decreases with the increasing of the cutting speed. Weber [87] studied the influence of different parameters such as the pulse energy and the repetition rate on heat accumulation through analytical models and gave the maximum number of pulses that can be tolerated to avoid thermal influence. Peter [88] performed cutting and punching experiments on CFRP using a nanosecond laser (maximum parameters: 7 MJ, 10 kHz, 30 ns), and obtained a good delamination-free cut seam using a high-speed scanning galvanometer fill processing mode. Jiao [89,90] used picosecond and nanosecond lasers with a wavelength of 532 nm to cut hole on CFRP plates. The effects of laser rotary cutting, parallel fill cutting and cross-fill cutting on the processing quality at different laser power were analyzed, and the results showed that the laser rotary cutting method had the highest removal efficiency, the smallest taper and the small thermal impact zone on CFRP (Figure 12).

In summary, comparing the CFRP machining quality with CW laser and pulsed laser, CW laser is 25% more efficient, but has a larger thermal effect on CFRP and results in a poor processing quality. So, CW laser is suitable for CFRP machining which require low processing accuracy and high machining efficiency. For pulsed lasers, the HAZ can be effectively reduced on the CFRP surface due to the continuous periodic cooling time between the adjacent pulses. So, it is suitable for the processing CFRP component which requires high-precision and low-damage. Comparing the nanosecond laser and the picosecond laser with different frequencies, it was found that the size of the HAZ decreases with decreasing of the time interval between adjacent pulses and the laser scanning speed. Therefore, a higher machining quality can be obtained for pulsed laser with a shorter pulse duration.

### 2.3. CFRP Field-Assisted Laser Processing Method

**Figure 13:**
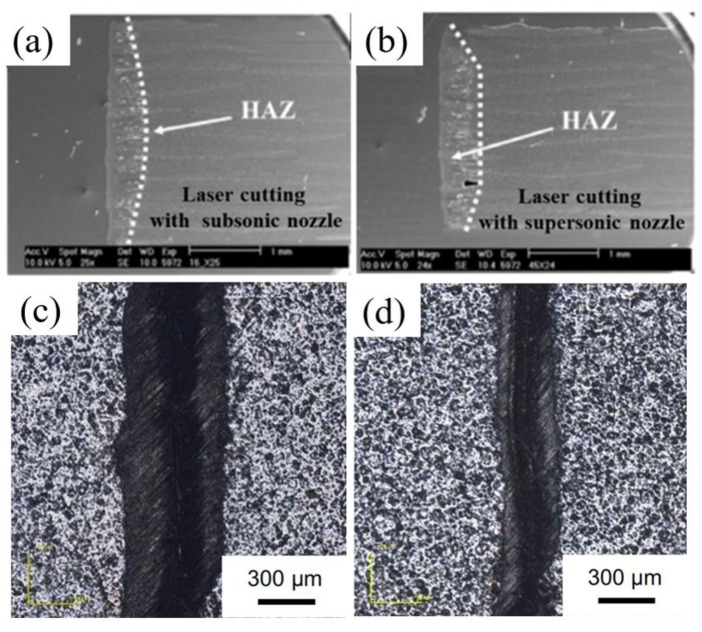
(**a**,**b**) HAZ of CFRP cutting with different Ar assistant methods Reprinted/adapted with permission from Ref. [91]; Copyright 2017, Elsevier. (**c**) HAZ of laser cutting; (**d**) HAZ of CFRP cutting with water jet guided laser processing technology Reprinted/adapted with permission from Ref. [97]. Copyright 2019, Springer Nature.

**Figure 14:**
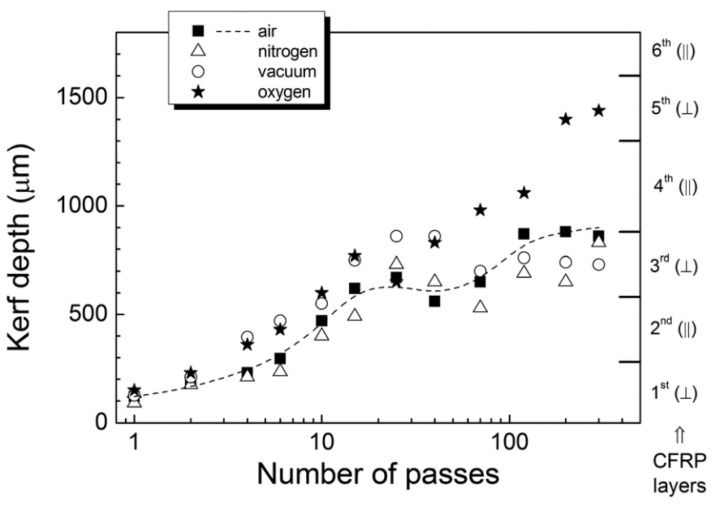
Evolution of kerf depth during multipass cutting of bidirectional CFRP in a chamber with different gas environment (F ¼ 28 J/cm^2^) Reprinted/adapted with permission from Ref. [94]. Copyright 2014, AIP Publishing.

Machining CFRP with energy field-assisted laser machining method can improve the cutting efficiency and quality. The commonly used auxiliary energy fields include airflow fields and water flow fields. Riveiro [91] used a 3 kW CO_2_ laser to cut a 3 mm thickness CFRP plate with off-axis supersonic nozzles and subsonic nozzles using Ar gas assistant (Figure 13a,b). It was found that the HAZ can be effectively reduced by using of auxiliary gases during the cutting process, but there are some long fibers on the cutting profile. Negarestani [92] found that adding oxygen into the inert gas can accelerate the decomposition of the resin matrix and promote material removal rate. At the same time, the HAZ can be reduced due to the nitrogen cooling in the cutting process. It concluded that the low oxygen volume fraction (typically 12.5%) and 0.8 MPa air pressure are the optimal parameter configurations to improve the quality of laser cutting of CFRP. Ramanujam et al. [93] made micropores on CFRP with carbon dioxide laser, and adjusted input parameters such as laser power, cutting speed and argon pressure. It was found that the cutting speed is the main factor affecting the hot dye area and the edge width, followed by the power. Kononenko et al. [94] used a picosecond laser to cut the depth multipass of bidirectional and unidirectional carbon fiber-reinforced plastics (CFRP). It is found that the auxiliary oxygen flow can significantly improve the cutting efficiency, and the oxygen support cutting can also solve the problem that occurs when cutting CFRP parallel to the fiber direction, where the angle appears strong deformation and widening (Figure 14).

**Figure 15:**
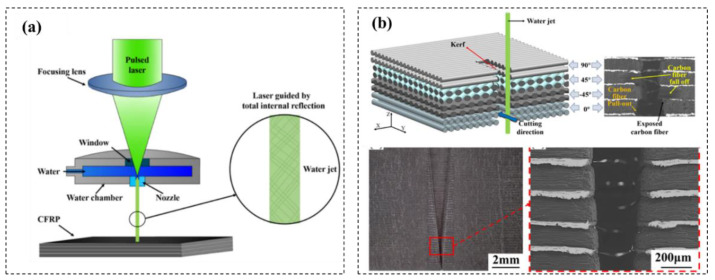
(**a**) Cutting CFRP with water jet guided laser processing technology; (**b**) CFRP cutting mechanism and cutting profile Reprinted/adapted with permission from Ref. [96]. Copyright 2021, Elsevier.

Water jet-guided laser processing technology combines the laser and the water jet, which can reduce the HAZ and increasing cutting depth in CFRP cutting process. Zhang [95] proposed a water jet-assisted cutting method of CFRP, which can greatly reduce the width of the cut slit and the HAZ comparing with the laser direct cutting method. It was found that the width of the seam on the upper surface increases first and then decreases with increasing of the flow rate of the water jet. The HAZ is smaller, and the morphology of the cutting inner wall is better than that of the laser direct cutting. Wu [96] investigated the influencing law of the process parameters such as laser power, CFRP feed speed and water jet speed on the cutting quality, and the influences of carbon fiber arrangement direction and laser cutting path on the CFRP cutting damage mechanism are analyzed (Figure 15). CFRP plates with the thickness of 1 mm, 2 mm, 4 mm and 10 mm CFRP is realized by adopting the parallel path layered scanning method.

Hua [98] cut CFRP with a 500 W millisecond Nd:YAG pulsed laser under water. The experiment results showed that the HAZ can be effectively reduced in CFRP laser cutting under water, which due to a large amount of heat can be absorbed by the water in the cutting process. Tangwarodomnukun et al. [98] studied the influence of laser transverse velocity, carbon fiber orientation, water flow rate and flow direction on cutting size and HAZ size. It is found that the use of high water flow rate can limit the expansion of HAZ and also help to remove materials. In addition, the water flow in the transverse direction of the laser can increase the cutting depth (Figure 13c,d). Sun et al. [99] uses three types of water jets to guide laser cutting CFRP. The study found that the characteristics of water jet guided laser cutting CFRP were significantly different from those of dry laser cutting in the case of multiple cutting with parallel path and without parallel path, while in the experiment using the multi pass scanning strategy without parallel path, the cornea was serrated on the side wall.

From the above analysis, it can be seen that in the CFRP laser machining process, water-assisted processing, underwater processing and gas-assisted methods can control the extraction of fibers and reduce the accumulation of residual heat in the processing area, so as to achieve a high machining quality.

## 3. CFRP Laser Processing Theoretical Model and Numerical Simulation

Due to the large difference between the matrix resin materials and carbon fibers in the physical properties, and the complexity of spatial composition for CFRP, it is difficult to understand the interaction mechanism between lasers and CFRP in machining process by experimental methods. So, it is helpful to analyze the interaction between materials and laser energy by using numerical simulation [100].

**Figure 16:**
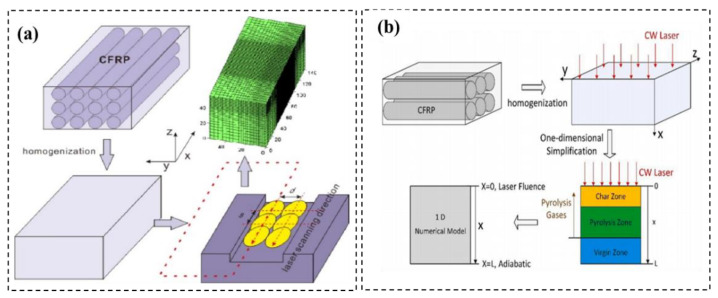
(**a**) Schematic diagram of laser milling and non-uniform mesh for the numerical model Reprinted/adapted with permission from Ref. [101]; Copyright 2017, Elsevier. (**b**) Schematic of volumetric ablation of CFRP composite with a CW laser and the corresponding one-dimensional numerical model Reprinted/adapted with permission from Ref. [102]. Copyright 2021, Elsevier.

Theoretical models of CFRP laser machining can lay a theoretical foundation for experiments and numerical simulations. Xu et al. [101] proposed a numerical model to study the material removal mechanism of CFRP laser grinding (Figure 16a). Through parameter analysis, it is found that the distance between two adjacent laser pulses with completely degenerated matrix should be used to effectively utilize mechanical erosion. Li et al. [102] proposed a one-dimensional transient model, which is based on the volume ablation of carbon fiber composites by continuous high power density lasers (Figure 16b). It was found that the higher the laser power density is, the smaller the pyrolysis zone is. Ge et al. [103] analyzed and compared the ultrasonic echo signals with different defect characteristics in CFRP and calculated the depth and length of the defect according to the time of longitudinal scattering wave (Lr) reaching the depth. It was found that the error of defect depth and length calculated by this method can be controlled within 7% and 5%. Chen et al. [104] used continuous wave fiber-reinforced laser to cut 2.0 mm thick carbon fiber-reinforced polymer laminate, established a modified thermal conductivity model, analyzed the heat transfer in unidirectional carbon fiber-reinforced polymer laminate, and compared with the experimental results, found that the model can be used for pretreatment.

Xu [101] studied the absorption behavior of CFRP on lasers and established a single-fiber surface absorption rate model by analyzing the refraction and reflection of lasers on the surface of uniaxial crystals. Genna [105] proposed a theoretical model that took into account the spatial distribution of the laser beam, the interaction time between the laser and the working material, the absorption coefficient, and the thermal properties of the material, and the cut seam width, the material removal rate, and the energy transmitted through the cut slit in CFRP laser cutting process were predicted. Sato [106] established the cauterization mass model and studied the dynamic energy change process in CFRP laser cutting. It was found that the laser energy was converted into heat, radiant energy, kinetic energy and ionization energy, and most of which was converted into ionization energy of oxygen and carbon. The model was established, and the ablation quality was predicted, the result showed that the ablation rate that calculated result (0.028 μg/pulse) is agree well with the experimental result (0.03 μg/pulse). Mucha [107] calibrated the temperature field in CFRP laser cutting by embedding thermal sensors in CFRP sample at different distances from the cutting seam, and dividing the HAZ into two different parts, which is a direct sublimation zone of the resin and a zone where the resin matrix is completely destroyed but not removed. A one-dimensional heat flow model was established, and the CO_2_ laser was used to cutting CFRP with different laser power and found that the error between the experimental data and theoretical model data is about 5%.

**Figure 17:**
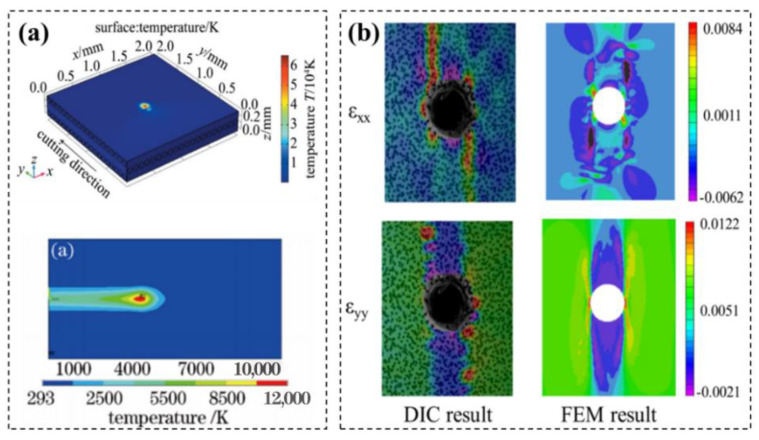
(**a**) Temperature distribution in CFRP laser cutting Reprinted/adapted with permission from Ref. [108]. Copyright 2019, Springer Nature.; (**b**) Tensile strength prediction in CFRP laser cutting Reprinted/adapted with permission from Ref. [109]. Copyright 2019, Springer Nature.

Based on the mathematical model of CFRP laser machining, lots of works were carried out by numerical method. Ohkubo [108] used the finite element volume method to perform a three-dimensional numerical simulation of the laser processing of CFRP, and the simulation results showed that the cross-sectional quality can be improved by adjusting the processing parameters and the appropriate value of carbon fiber and resin removal speed was achieved (Figure 17a). Li [109] established the progressive damage failure mode of open-hole CFRP laminate based on the two-dimensional Hashin failure criterion by using ABAQUS finite element software, and the results showed that the full-field stress distribution is non-uniform and asymmetrical, and the crack propagation and failure modes are consistent with the development of high-level strain around the hole. At the same time, it was also found that the full-field strain of CRRP plates is closely related to tensile load level and fiber orientation, and the numerical model results is consistent with the experimental results well (Figure 17b).

Hou [110] defined laser heat source models in the thermal analysis process considering three types of boundary conditions by using the ANSYS software, and the influence of the laser power, the laser scanning speed and the laser spot radius on the temperature that along the thickness direction of carbon fibers and perpendicular to the laser scanning direction were analyzed. The relationship between laser cutting process parameters and temperature was obtained, which provided a theoretical guidance for laser heat propagation and control of thermal impact zone. Di [111] established a multiphysics model of laser cutting carbon fiber composites by using COMSOL software based on the anisotropy characteristics of carbon fiber composites. A three-dimensional temperature field distribution was obtained, as well as the transmission law of heat in fibers and resins and the influence of laser parameters on cutting quality was predicted.

Ohkubo [112] performed numerical simulations on CFRP laser cutting, which is helpful to understand the generation mechanism of the HAZ and improve the cutting quality. Li [113] summarized the surface defects and suppression methods of CFRP laser processing, and took femtosecond laser processing CFRP as an example, a process scheme for improving processing quality and efficiency is proposed through the combination of theory, simulation and experiment analysis. Yu [114] studied the influence of the laying direction and resin content of carbon fibers on the direction of laser energy transmission and the cutting quality, and established a three-dimensional finite element model in which the resin content increased from 30% to 50% when the laying angle of carbon fiber was 0°, 45°, and 90°, respectively. It was found that the fiber laying angle influenced the temperature field and the HAZ width and maximum temperature show approximate linear changes with the increase of resin content. Furthermore, comparing the numerical result and the experimental results, the average error of the surface carbon fiber ablation width in the numerical simulation results is 10.66%, and the average error in the HAZ width is 13.09%.

Liu [115] established a three-dimensional thermal calculation model of carbon fiber composite plate based on the theory of thermodynamics, the temperature field and stress field of carbon fiber composite material under laser irradiation were predicted. The results showed that the temperature rise trend of laser irradiation was consistent with the change of the temperature reduction trend, which was basically consistent with the experimental temperature results. Cao [116] investigated the bottom depression phenomenon in the blind hole processing with the picosecond pulsed laser. The numerical simulation was carried out by using COMSOL software, and the effects of segmented variable speed and linear variable speed scanning speed strategies on the machining depth, thermal impact zone and bottom micromorphology of blind holes were studied.

**Figure 18:**
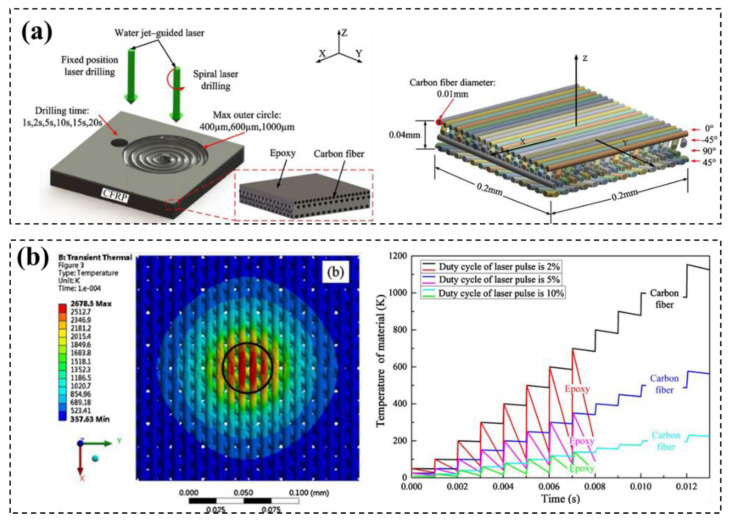
Numerical model (**a**) and temperature prediction (**b**) of CFRP cutting with waterjet assisted laser technology Reprinted/adapted with permission from Ref. [117]. Copyright 2020, Elsevier.

Based on the “element birth and death” technique in the finite element method, the three-dimensional transient temperature field of heterogeneous fiber matrix and the subsequent material removal model are established by Zhang [117]. Under this model, the influence of the duty cycle of laser pulse on the temperature field distribution of materials was studied, and the model was verified by experiments under the same process conditions (Figure 18). The results showed that CFRP composites processed by water jet-guided laser processing have obvious advantages over traditional laser processing. Adjusting the duty cycle of laser pulses affects the shape and temperature distribution of the composites after drilling.

In summary, machining CFRP with lasers is a complex thermos-physical process. The carbon fibers and resins will rapidly absorb laser energy as the laser irradiation on the CFRP plate, and the resin matrix material melts firstly, and then the carbon fiber material continues to absorb the heat and transfer the heat into the material, thereby forming the cut slit and the HAZ. From the melting of the matrix material to the transfer of heat inside the material, many disciplines such as materials science, chemistry and thermodynamics are involved, as well as a series of influencing factors such as the laser pulse width, the laser scanning speed, the laser power and the basic properties of the CFRP material have impacts on the internal heat transfer and the HAZ formation. Therefore, the relevant research considering the characteristics of laser and the anisotropy of CFRP, the numerical multi-physics model was established, and the simulation analysis was carried out by using finite element software such as ANSYS, ABAQUS and COMSOL, to predict the cut seam width and the thermal impact zone with different laser parameters and material parameters, which can improve efficiency and guides the experiment effectively.

## 4. Conclusions

With the increasing application of CFRP in industry and the developing of laser machining technology, using of lasers to drill, cut and precision machine CFRP composites becomes more and more important in lightweight manufacturing. However, due to the anisotropy and heterogeneity characteristic of carbon fiber composites, lots of processing problems such as delamination, burrs and thermal damage during processing was produced in machining process. On the basis of summarizing and sorting out different CFRP processing methods such as mechanical machining, waterjet processing, ultrasonic vibration machining, the defects in different processing methods were pointed out, and the research on process optimization and numerical simulation of laser machining CFRP was reviewed systematically, and some conclusions and the development trends of CFRP laser cutting technology are draw as below.

(1) Comparing the CFRP cutting quality with CW laser and pulsed laser, it can be found that it has a higher processing efficiency but a larger thermal effect for CW laser. So, CW laser is suitable for CFRP cutting which require low processing accuracy and high cutting efficiency. Different scanning strategies (process optimization) can be used in the future to process carbon fiber composites with high efficiency and high quality. For pulsed lasers, the HAZ can be effectively reduced on the CFRP surface due to the continuous periodic cooling time between the adjacent pulses. So, it is suitable for the machining CFRP component which requires high-precision and low-damage. Moreover, the selection of suitable laser parameters (pulse width, frequency, scanning path, etc.) is conducive to the cooling of materials in the processing process and improves the processing quality.
(2) CFRP has different absorption coefficient for lasers with different wavelength. For the infrared laser, the polymer matrix absorption rate of laser is less than 15%, and 85% of the energy pass through the polymer matrix to heat the carbon fibers directly. On the other hand, the UV laser energy is absorbed by the resin matrix almost completely. To this end, the CFRP material removal mechanism is different for these two types of lasers, and the UV laser has a better cutting edge quality than the IR laser, and the HAZ is larger for IR laser than that for UV laser. In this regard, in the future, we can use high-power ultraviolet lasers for high-precision, low-damage CFRP cutting.
(3) Cutting CFRP with energy field assistant laser machining method can improve the cutting efficiency and quality. Summarizing the common auxiliary means in the laser processing process, such as water-assisted laser processing, gases-assisted laser processing and underwater processing. All of these techniques can control the extraction of fibers and reduce the accumulation of residual heat in the processing area, so as to achieve a high cutting quality. In the future, other energy filed can be introduced in CFRP laser cutting to improve the quality and efficiency such as magnetic field, electric field, ultrasonic field, flow field and external force field, which needs further investigated.
(4) To understand the mechanism of CFRP laser cutting and with lasers more clearly and obtained the temperature distribution in cutting process, the numerical multi-physics model was established, and the simulation analysis was carried out by using finite element method. The cut seam width and the thermal impact zone with different laser parameters can be predicted, which providing a certain theoretical and experimental basis for laser cutting of CFRP. However, the numerical analysis of material removal physical process of CFRP under the action of short pulse laser is still lacking, especially the simulation of temperature field, stress field and material removal process under the action of ultrashort pulse laser, such as femtosecond laser and picosecond laser, which needs further research.

## Data Availability

Not applicable.
